# Anti-Vaccinationists and Modified Smallpox

**Published:** 1909-08-14

**Authors:** John H. Bonner

**Affiliations:** Leicester


### Editor's Letter-Box.

### ANTI-VACCINATIONISTS AND MODIFIED SMALLPOX.

*To the Editor of* The Hospital.

Sir,—I have read with much interest your article in the issue of July 31 bearing the above title. With the first part I have no desire or intention to deal, beyond remarking that the anti-vaccinators do not monopolise the opprobrious epithets used towards opponents on this subject. It must not be overlooked that the profession have obtained penal powers for the enforcement of a surgical operation, and that resistance thereto has cost those who disbelieve in it many thousands of pounds, whilst many more have had their homes sold up or gone to gaol—only to be dubbed cranks, eccentrics, faddists, fanatics, etc. The elucidation of a great problem does not lay that way. But the writer of your special has made a slip when, after quoting Dr. Millard to the effect that it is "the adult population which is the principal factor in the spread of the disease," he adds by way of comment "that part of the population, in fact, which is least efficiently vaccinated." Obviously, according to the pro-vaccinist theory, the least protected, of whatever age, are those who have never been vaccinated at all, and yet in this borough—with tens of thousands such—they exhibit no particular susceptibility either to contract or to spread the fever. I quite appreciate the difficulty of diagnosing accurately in every instance smallpox in its earlier stages, but the point is that modified attacks are capable of communicating virulent forms to others. That seems to show that the severity of an attack is regulated by the bodily, and not by the vaccinal, condition of each individual. For something like thirty years—with an ever-increasing unvaccinated population—the comparative immunity of the latter class has continued most marked, despite the severe tests to which the town has been put during smallpox prevalence, up and down the country, and even during the epidemic periods of 1892-3 and 1903-4 the abnormally low case-fatality must have confounded those who have constantly predicted what will happen here whenever smallpox is imported. In the former instance 346 cases produced 21 deaths, whilst on the latter occasion, with 715 cases there were only 25 deaths. Does not this suggest that there are other modifying influences at work besides that of vaccination? I venture to think that the production of a healthier race of children in a town where the value of sanitation is appreciated accounts for a good deal, coupled with the more rational treatment of patients now as compared with olden times. You tell us what the profession are prepared to do "when the biology of smallpox has been worked out," but I doubt not will admit that this—one of the oldest recorded diseases—still baffles your ranks. Less than three weeks ago it was again reported (this time from Rio de Janeiro) that the microbe had been found. Periodically we get a statement similar to this from various parts of the world, only to be apprised shortly afterwards that it was not the genuine article after all. By all means continue your scientific investigations, but drop your compulsion and the association of the noble work of the profession with that of the policeman, the bailiff and the warder, whilst you are still in the experimental stages.—Sincerely yours,

Leicester, August 9, 1909. John H. Bonner.

Mr. Leonard Colebrooke, M.B., B.S. London, has been appointed resident medical officer of the newly opened inoculation wards in the Clarence Wing at St. Mary's Hospital.

